# Assessment of occupational hazards, health effects, and personal protective equipment usage among motorcycle food delivery riders in Thailand: a cross-sectional survey

**DOI:** 10.1186/s12995-025-00460-x

**Published:** 2025-05-10

**Authors:** Siriaran Kwangsukstith, Vithawat Surawattanasakul, Chollada Mahakkanukrauh, Jinjuta Panumasvivat, Wachiranun Sirikul

**Affiliations:** 1 Faculty of Medicine, Chiang Mai University, Chiang Mai, 50200 Thailand; 2 Department of Community Medicine, Faculty of Medicine, Chiang Mai University, 110 Intawaroros Road, Sri Phum Subdistrict, Muang, Chiang Mai, 50200 Thailand; 3 Environmental and Occupational Medicine Excellence Center, Faculty of Medicine, Chiang Mai University, Chiang Mai, 50200 Thailand

**Keywords:** Motorcycle food delivery riders, Occupational hazards, Health effects, Personal protective equipment

## Abstract

**Background:**

Motorcycle Food Delivery Riders (MFDRs) play a vital role in the online food delivery industry, yet their prolonged time on the road exposes them to numerous occupational hazards, health risks, making them more vulnerable than both regular motorcyclists and the general population. The study aimed to investigate the working conditions, occupational hazards, health issues, use of personal protective equipment (PPE), and the association between occupational hazards and health effects among Thai MFDRs.

**Methods:**

A cross-sectional study was conducted from November 2021 to mid-February 2022 in Chiang Mai, Thailand, using an anonymous self-reported survey. A total of 709 MFDRs participated in the survey. Data were collected on background information, job characteristics, occupational hazards, health issues, and the use of PPE over the previous six months. The data were analyzed using a multivariable logistic regression model.

**Results:**

Most MFDRs are frequently exposed to physical hazards, including heat and sunlight (91.6%), as well as chemical hazards such as exhaust smoke (90.1%) and particulate matter (PM) (86.1%). Most MFDRs reported suffering from musculoskeletal disorders (62.1%) and eyes-related problems (45.1%). The most common problems were shoulder pain (39.2%), neck pain (38.1%), and burning eyes (33.3%). Multivariable logistic regression analysis indicates that biomechanical hazards are significantly associated with musculoskeletal disorders (MSDs) (*p* < 0.05), while psychological hazards significantly associated with headaches, insomnia, and feeling depressed (*p* < 0.05). Additionally, physical hazards such as sunlight and heat, noise, and whole-body vibrations, are also significantly associated with headaches, flu-like symptoms, and insomnia (*p* < 0.05). The most worn PPE by the participants were helmets (99.72%), long-leg pants (99.72%), masks (99.29%), and thermal jackets (98.17%).

**Conclusions:**

The high prevalence of occupational hazards and health issues among MFDRs is worrisome. It is critical for platform companies and health sectors to introduce effective protective measures for workers, including establishing health surveillance, and supplying PPE.

**Supplementary Information:**

The online version contains supplementary material available at 10.1186/s12995-025-00460-x.

## Background

Over the past decade, the number of food delivery platforms has increased more than fivefold, resulting in a substantial rise in the number of food delivery workers [1]. One key factor contributing to this growth is the implementation of social distancing measures during the COVID-19 pandemic, which were aimed at minimizing physical contact [2]. This trend is expected to continue expanding steadily, even in the post-pandemic period [3]. Since there are large population of motorcycle food delivery riders (MFDRs) worldwide, their health constitutes a significant public health concern. Their works frequently takes place outdoors and, on the road, thus they are susceptible to various hazards, injuries, accidents, health effect and environmental conditions such as weather and pollution [4, 5].

MFDRS in Thailand are exposed to five types of health hazards: physical, chemical, biological, psychological, and biomechanical hazards. First, physical hazards, including noise, whole-body vibration, and heat and sunlight. Noise from traffic and earphones can lead to tinnitus, headaches, eyestrain, sleep disturbances [6]. Whole-body vibration from motorcycle engines and poor road conditions may contribute to musculoskeletal disorders [7]. Insufficient protection from heat and sunlight due to inadequate personal protective equipment (PPE) can result in skin burns and eye damage, such as senile cataracts and skin cancer [8–10]. Second, participants are also exposed to chemical hazards, such as particulate matter (PM) and traffic-related smoke from vehicle exhaust. PM 2.5 and PM 10 are often generated by open burning in industrial areas, agricultural areas such as biomass burning. Acute exposure of these chemical hazards can lead to cough, wheezing, and exacerbation of asthma [11]. Chronic exposure may result in serious respiratory and cardiovascular conditions, including lung cancer, myocardial infarction, dermatitis [11]. Third, biological hazards, this category involves bacterial and viral transmission from helmets [12], and food packaging [13] handled by vendors and customers. It can lead to upper respiratory infections from influenza and COVID-19 [5, 13, 14]. Fourth, biomechanical hazards, which arise from prolonged or inappropriate postures and repetitive movements during service. Such conditions can cause pain in the upper extremities [15] due to improper posture during motorcycle rides. Lastly, psychological hazard, the stress particularly from customer behavior, long shifts, income uncertainty, and the competitive environment, can lead to poor mental health, sleep deprivation, depressive symptoms [16].

To mitigate the risk against various occupational hazards, delivery riders often rely on Personal Protective Equipment (PPE) [17]. The main PPE used include helmets for head protection, reflective vests (fluorescent) to enhance visibility in low-light conditions, gloves to shield hands from cuts and abrasions, and protective eyewear to guard against sunlight and debris [18]. Overall, an understanding of gig riders’ health especially MFDRs is crucial for the development of their health promotion and safety. Given the rapid expansion of food delivery industry and the unexplored hazards and health effects faced by riders, there is a notable gap in research on the association between these factors. Therefore, this study was initiated to examine the prevalence of occupational hazards, the health effects, and the use of personal protective equipment (PPE) among MFDRs in Thailand, as well as the association between occupational hazards and the health effects experienced by these riders.

## Methods

This cross-sectional study is reported according to STROBE (strengthening the reporting of observational studies in epidemiology) guidelines [19].

### Study design, setting and study participants

This cross-sectional study was conducted among MFDRs working in the city of Chiang Mai, Thailand from November 2021 to mid-February 2022 via an anonymous online survey. To ensure respondent anonymity, the survey included confidentiality statements and refrained from collecting personally identifiable information. Participants received a unique link of questionnaire, and authorized personnel adhered to strict data handling procedures. The inclusion criteria were individuals who are older than 18 years old, had been working as full-time food delivery riders for a minimum of six months at the time of the study. To engage a diverse group of participants, improve the response rate, and ensure the relevance of the findings, a variety of recruitment strategies were employed, both online and offline. These included distributing flyers, posting posters, and utilizing online platforms like Facebook groups and LINE OpenChat for bi-weekly announcements. By using multiple recruitment channels, the study aimed to reach a wider and more representative sample of MFDRs, thereby enhancing the study’s validity. A total of 1,028 participants accessed the online platform, with 709 participants (68.9%) providing complete responses to all the survey questions.

### Questionnaires

This self-reported survey, conducted as part of a broader project, used a convenient sampling method [20]. A standardized, validated questionnaire was prepared by the research team with experts’ opinions based on the observation, experience, and previous literatures. The questionnaire was tested for validity by two independent experts from public health and occupational medicine specialties. Then the pilot study was conducted among 30 motorcycle taxies to test- retest the reliability. The reliability test was performed by using Cronbach’s alpha analysis. The value of reliability test on “Occupational hazards”, “New onset health effects” and “PPE” was 0.92, 0.85, and 0.89, respectively indicating a high level of reliability.

The questionnaire consisted of five main parts, sixty-one questions.

1. Socio-demographic information (11 questions): This section collected sex, age, educational level, weight, height, marital status, smoking status, alcohol drinking, annual health check-up, accident insurance, and routine vehicle maintenance and check-up.
2. Job characteristics (5 questions): This section collected working hours per week, working experiences, orders per hour, income per day, and work shift.
3. Occupational hazards (14 questions): The occupational hazards are separated into 5 main categories: physical, chemical, biological, biomechanical, and psychosocial hazards. This section used a Likert scale with five levels (always, usually, sometimes, seldom, never) to access the frequency of the occupational hazard. From Likert scale, participants who reported responses of “always”, or “usually” were categorized as exposed to the hazards, while those reporting, “sometimes”, “seldom” or “never” were classified as not exposing to the hazards.
4. New onset health effects (22 questions): This section collected information about occurrence of new-onset and exacerbation of health problems experienced by the participants. The questionnaire focused on different parts of the body, namely musculoskeletal disorders (MSDs), eyes, respiratory system, skin, and others. This section used a yes/no question to access the frequency of the new onset health effects or exacerbation of health problems during the past 6 months.
5. PPE (9 questions): The PPE listed in the questionnaire includes: long pants, helmets, face masks, thermal jackets, boots, gloves, sunglasses or wind goggles (protective eyewear), earplugs, and knee, arm, or trunk pads. This section used a Likert scale with five levels (always, usually, sometimes, seldom, never) to access the frequency of the rider’s use. From Likert scale, participants who reported responses of “always”, or “usually” were categorized as using the PPE, while those reporting, “sometimes”, “seldom” or “never” were classified as not using the PPE. The questionnaire items are in Supplementary file 1.

### Statistical and data analysis

The survey data obtained from the web-based platform were analyzed using STATA software version 16.0 (Stata Corp., College Station, TX, USA). Descriptive statistics were analyzed, including frequency (n), percentage (%), mean, median, interquartile range (IQR) standard deviation (SD), percentile 25th (P25), and percentile 75th (P75). Multiple-adjusted logistic regression was analyzed to determine association between occupational hazards with health effects among MFDRs. Variables used in multivariable logistic regression model were age, sex, working hour per day, and additional variables based on each hazard. Adjusted odds ratio (aOR) and 95% confidence interval (CI) have been used to quantify the association between variables. Variables that had differences were set significant for *p* < 0.05.

## Results

### Socio-demographic and job characteristics of motorcycle food delivery riders in Thailand

The general and occupational characteristics of the participants are presented in Table 1. A total of 709 MFDRs participated in this survey. Most participants were male 68.7%. The participants reported working at 54 h per week (IQR = 28.00), receiving median of 3 orders per hour (IQR = 1.00) and had daily income of approximately 477.6 Baht (IQR = 287.2) (Approximately 14.3 USD (IQR = 8.60)). Majority of the participants reported working at daytime (80.7%) and had working experience of 2 years (IQR = 2.00).

**Table 1:** Socio-demographic and job characteristics of MFDRs, Thailand. (*N* = 709)

Variables	*n* (%)
Sex	
Male	487 (68.7)
Female	222 (31.3)
Age	
≤ 20	36 (5.1)
21–35	403 (56.8)
36–45	218 (30.7)
≥ 46	52 (7.3)
Education level	
Primary School or less	35 (4.9)
Secondary School	325 (45.8)
Diploma	110 (15.5)
Bachelor’s degree or higher	239 (33.7)
BMI	
Underweight (< 18.5)	33 (4.7)
Normal (18.5–22.9)	221 (31.2)
Overweight (23-24.9)	119 (16.8)
Obese (≥ 25)	336 (47.4)
Marital Status	
Single	469 (66.1)
Married	192 (27.1)
Divorced/Widowed	48 (6.8)
Current Smoking	183 (25.8)
Current Drinking	337 (47.5)
Working hour/ week (hours), median (IQR)	54 (28)
Working experience (year), median (IQR)	2 (2)
Orders per hour (order), median (IQR)	3 (1)
Daily Income (Baht^a^) median (IQR)	477.6 (287.2)
Shift	
Day Shift	572 (80.7)
Night Shift	137 (19.3)
Annual health check-up	287 (40.5)
Has accidental insurance	437 (61.6)
Routine vehicle maintenance	646 (91.1)

^a^The exchange rate at the time of the study (33.4 Baht = 1 USD)

### Prevalence of occupational hazards exposed by the MFDRs

**Fig. 1:**
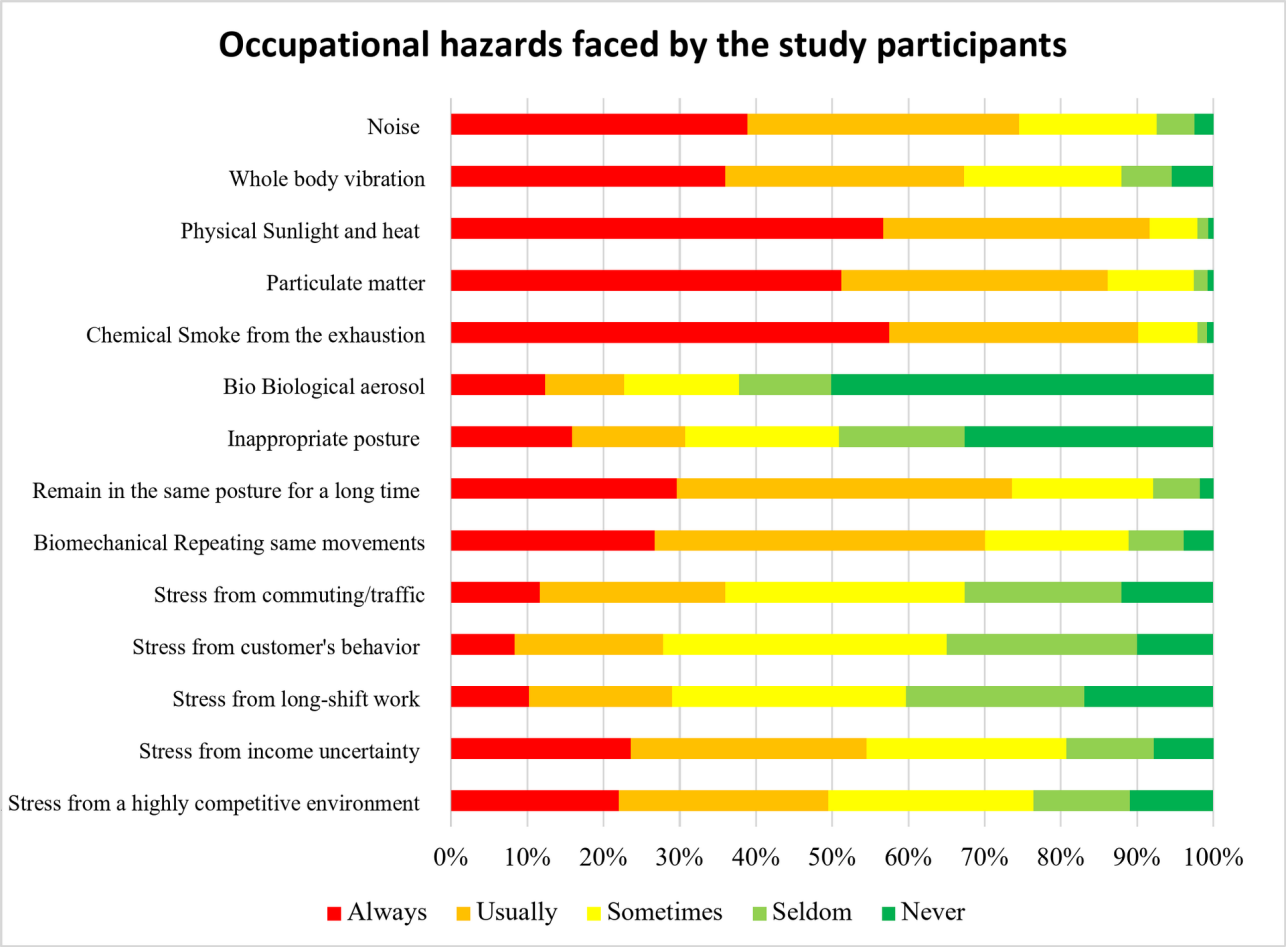
Prevalence of occupational hazards faced by the study participants

Figure 1 details the acquired occupational hazard exposure. Most participants were frequently exposed to physical hazard and chemical hazard. Almost all participants exposed heat and sunlight, exhaustion smoke, and PM 2.5 for 91.6%, 90.1% and 86.1% respectively. This were followed by noise (74.5%) and whole-body vibration (67.3%), which around three-fourth of the participant faced every day in their carrier. In addition, they also suffered with some biomechanical hazard, for instance around 70% of the participants suffered remaining in the same posture for a long time (73.6%) and repetitive motions (70.0%). For psychological hazards, dealing with stress and emotions, stress form income uncertainty and highly competitive environment were the main concerns of 54.5% and 49.5% respectively. Others psychological hazard such as stress from commuting and traffic (36.0%), stress from customer’s behavior (27.8%) and stress from long-shift work (29%). Interestingly, the biological hazard from biological aerosol was considered least problematic with only 22.7% complaining and around half of the participants (50.1%) never exposed to biological aerosol. 

### Prevalence of health effects among MFDRs

**Fig. 2:**
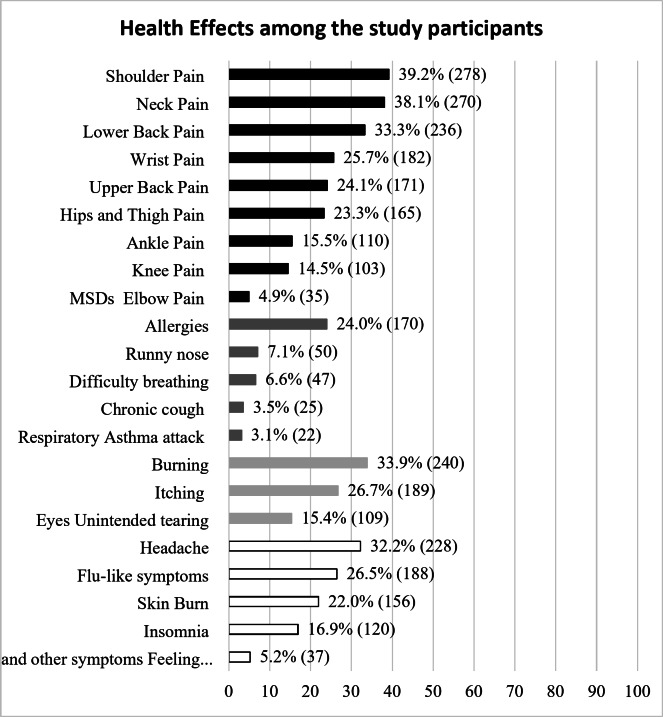
Prevalence of health effects among MFDRs

A result on prevalence of new onset occurrence and exacerbation of health problems after working as MFDRs was shown in Fig. 2. The most common health problems reported from the participants was work-relatedness musculoskeletal disorders (WRMSDs) (62.1%). Followed by eye-related problems (45.1%), respiratory-related problems (29.1%). The most common MSDs that were reported the most are as follows: shoulder pain (39.2%), neck pain (38.1%) and lower back pain (33.3%). The most common eyes-related problems that occurred are burning eyes (33.9%), followed by itching eyes (26.7%) and unintended tearing (15.4%). For respiratory-related problems, allergies are the most striking health that effects of nearly one quarter of the respondents being reported (24.0%). Additionally, Headache (32.2%), flu-like symptoms (26.5%), skin burn (22.0%), insomnia (16.9%) and feeling depressed (5.2%) were others common health effects experienced by the MFDRs.

### Association of biomechanical hazards with work-relatedness musculoskeletal disorders among MFDRs

The results of the multivariable logistic regression analysis regarding the association between biomechanical hazards and the prevalence of WRMSDs among MFDRs are presented in Table 2. After adjustment of potential confounders such as age, sex, working hours per day, and BMI, the results showed that exposure to three out of four biomechanical hazards, including static positions, repetitive movements and whole-body vibration were highly significantly associated with eight out of nine WRMSDs (neck pain, shoulder pain, wrist pain, upper back pain, lower back pain, thigh pain, knee pain, ankle pain) (*p* < 0.001) and significantly associated with elbow pain (*p* < 0.05). While exposure to awkward position was significantly associated with only six out of nine MSDs, excluding shoulder pain, lower back pain, and knee pain (*p* < 0.05).

**Table 2:** Association between Biomechanical hazards and work-related musculoskeletal disorders among MFDRs

Health Effect	Exposures							
	Awkward Positions		Static Positions		Repetitive Movements		Whole body vibration	
	aOR (95% CI)	*p*-value	aOR (95% CI)	*p*-value	aOR (95% CI)	*p*-value	aOR (95% CI)	*p*-value
Neck	1.60 (1.15,2.22)	0.005*	3.60 (2.39,5.43)	< 0.001**	2.70 (1.87,3.90)	< 0.001**	2.75 (1.92,3.94)	< 0.001**
Shoulder	1.28 (0.92,1.77)	0.147	3.37 (2.26,5.03)	< 0.001**	2.89 (2.00,4.17)	< 0.001**	2.26 (1.60,3.20)	< 0.001**
Elbow	2.69 (1.33,5.47)	0.006*	3.01 (1.03,8.81)	0.044*	7.62 (1.79,32.39)	0.006*	4.94 (1.48,16.46)	0.009*
Wrist	1.65 (1.15,2.37)	0.007*	3.62 (2.20,5.95)	< 0.001**	3.35 (2.12,5.30)	< 0.001**	3.42 (2.20,5.32)	< 0.001**
Upper back	1.67 (1.16,2.42)	0.006*	3.75 (2.23,6.29)	< 0.001**	2.54 (1.64,3.95)	< 0.001**	2.74 (1.77,4.25)	< 0.001**
Lower back	1.21 (0.86,1.70)	0.275	3.12 (2.04,4.76)	< 0.001**	2.97 (2.00,4.41)	< 0.001**	2.22 (1.54,3.20)	< 0.001**
Thigh	1.68 (1.16,2.43)	0.006*	4.24 (2.45,7.34)	< 0.001**	2.36 (1.52,3.67)	< 0.001**	3.40 (2.14,5.40)	< 0.001**
Knee	1.52 (0.98,2.37)	0.062	4.45 (2.19,9.05)	< 0.001**	3.34 (1.82,6.14)	< 0.001**	1.83 (1.11,3.02)	0.017*
Ankle	1.74 (1.13,2.66)	0.011*	4.80 (2.36,9.75)	< 0.001**	3.74 (2.03,6.87)	< 0.001**	3.24 (1.85,5.68)	< 0.001**

The association between biomechanical hazards and health effects among MFDRs was identified by multivariable logistic regression analysis with adjustment for confounders, including sex, age, working hours per day and body mass index. * Significant association at *p *< 0.05 ** Highly significant association at *p* < 0.001

### Association between psychological hazards and health effects among MFDRs

After adjustment of potential confounders such as age, sex, working hours per day, sleep hours per day and income per day, the results, as presented in Table 3, showed that exposure to four psychological hazards, including stress from commuting/traffic, stress from long-shift work, stress from income uncertainty and stress from a highly competitive environment were significantly associated with headache, insomnia, and feeling depressed (*p* < 0.05). While stress from customer behavior were significantly associated with headache and insomnia, but except for feeling depressed.

**Table 3:** Association between psychological hazards and health effects among MFDRs

Health Effect	Exposures									
	Stress from commuting/traffic		Stress from customer behavior		Stress from long-shift work		Stress from income uncertainty		Stress from a highly competitive environment	
	aOR (95% CI)	*p*-value	aOR (95% CI)	*p*-value	aOR (95% CI)	*p*-value	aOR (95% CI)	*p*-value	aOR (95% CI)	*p*-value
Headache	2.43 (1.74,3.38)	< 0.001**	1.84 (1.30,2.62)	< 0.001**	2.05 (1.45,2.90)	< 0.001**	1.98 (1.42,2.76)	< 0.001**	1.87 (1.35,2.60)	< 0.001**
Insomnia	2.93 (1.95,4.40)	< 0.001**	1.78 (1.18,2.70)	0.007*	3.13 (2.08,4.71)	< 0.001**	3.42 (2.16,5.44)	< 0.001**	2.72 (1.77,4.17)	< 0.001**
Feeling Depressed	2.85 (1.42,5.71)	0.003*	1.49 (0.74,3.00)	0.270	2.90 (1.47,5.74)	0.002*	3.6 (1.55,8.37)	0.003*	2.34 (1.13,4.86)	0.023*

The association between psychological Hazards and health effects among MFDRs was identified by multivariable logistic regression analysis with adjustment for confounders, including sex, age, working hours per day, sleep hours per day and income per day. * Significant association at *p *< 0.05 ** Highly significant association at *p *< 0.001

### Association between physical hazards and health effects among MFDRs

After adjusting for potential confounders such as age, sex, working hours per day, PM2.5, and work shift, the results presented in Table 4 show that exposure to sunlight and heat was significantly associated with burning eyes, itching eyes, sunburn, headache, flu-like symptoms, and insomnia (*p* < 0.05). Additionally, exposure to noise and whole-body vibration (after adjustment for confounders such as age, sex, and working hours per day) was significantly associated with headache, flu-like symptoms, and insomnia (*p* < 0.05). It should be noted that all three exposures; noise, whole body vibrations, sunlight and heat were highly significantly associated to headache (*p* < 0.001).

**Table 4:** Association between physical hazards and health effects among MFDRs

Health effects	Exposures					
	Sunlight and heat ^a^		Noise ^b^		Whole body vibrations ^b^	
	a OR (95% CI)	*P*-value	a OR (95% CI)	*P*-value	a OR (95% CI)	*P*-value
Burning eyes	3.12 (1.48, 6.57)	0.003*	N/A	N/A	N/A	N/A
Itching eyes	2.29 (1.08, 4.84)	0.031*	N/A	N/A	N/A	N/A
Unintended tearing	1.95 (0.75, 5.01)	0.168	N/A	N/A	N/A	N/A
Sunburn	3.09 (1.20, 7.97)	0.020*	N/A	N/A	N/A	N/A
Headache	4.18 (1.83, 9.51)	< 0.001**	2.12 (1.42, 3.16)	< 0.001**	2.81 (1.92, 4.12)	< 0.001**
Flu-like symptoms	2.62 (1.20, 5.73)	0.016*	1.63 (1.08, 2.46)	0.020*	2.09 (1.41, 3.10)	< 0.001**
Insomnia	4.06 (1.23, 13.40)	0.022*	2.71 (1.53, 4.81)	0.017*	3.64 (2.09, 6.34)	< 0.001**

The association between noise, whole body vibrations, sunlight and heat exposures, and health effects among MFDRs was identified by multivariable logistic regression analysis with adjustment for confounders, including ^a^sex, age, working hours per day, work shift and PM2.5 and ^b^ sex, age and working hours per day. * Significant association at *p *< 0.05 ** Highly significant association at *p *< 0.001

### The use of personal protective equipment among MFDRs

**Fig. 3:**
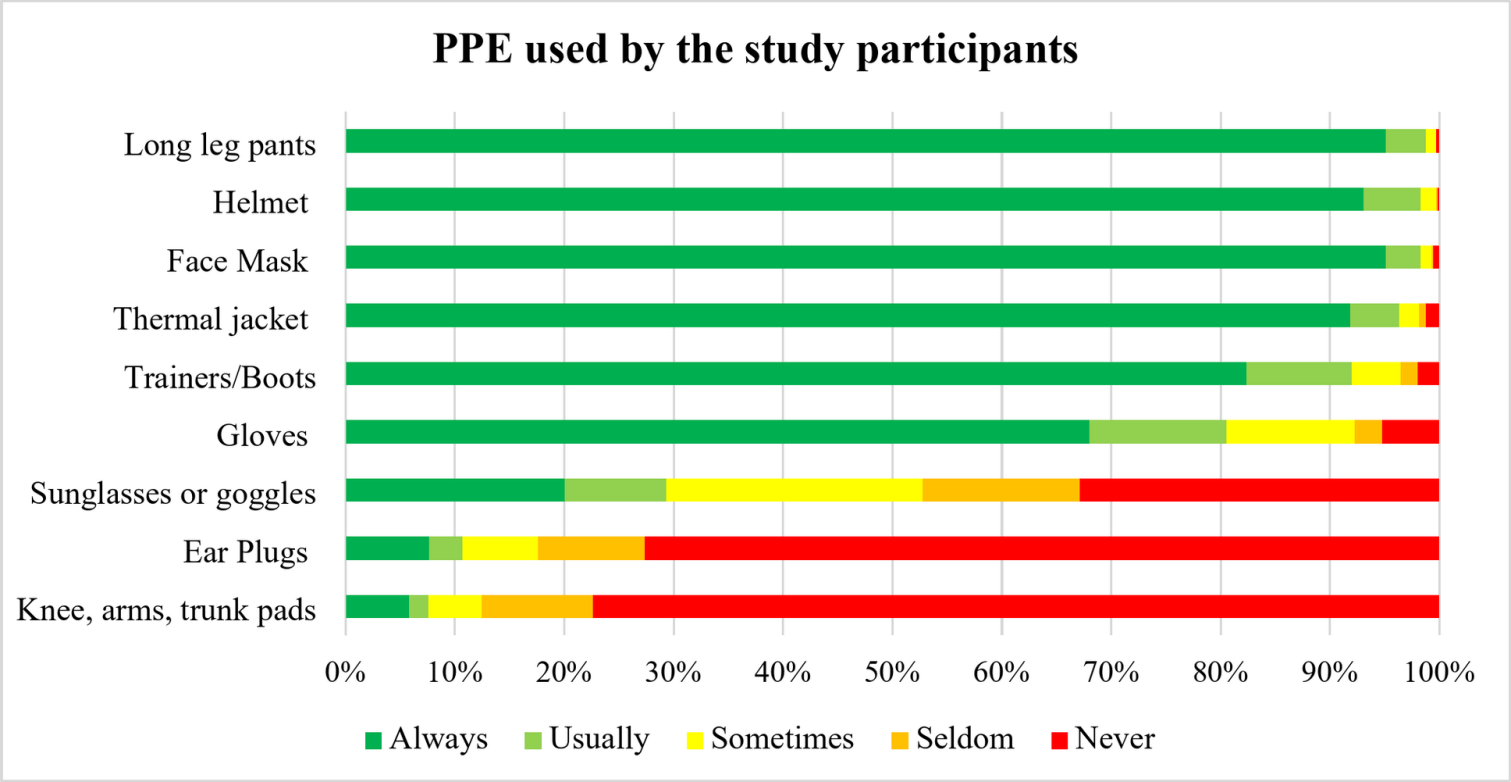
Prevalence of personal protective equipment (PPE) used among MFDRs

As shown in Fig. 3, most Thai MFDRs used variety of personal protective equipment (PPE) while working. The most employed PPE were long leg pants and helmets, both were worn by almost all the participants (99.72%). This was followed by face masks (99.29%) and thermal jacket (98.17%). Other PPE worn by the participants included boots (96.47), gloves (92.24%), sunglasses or wind goggles (protective eyewear) (52.75%). Interestingly earplugs and knee-arm-or trunk pads were utilized by only 17.63% and 12.48% of participants, respectively.

## Discussion

This study aimed to investigate the prevalence of occupational hazards, health effects, and the use of PPE among MFDRs. We conducted an analysis focusing on the association between occupational hazards and health. The majority of MFDRs frequently encounter physical hazards, with heat and sunlight being the most prevalent (91.6%). Additionally, chemical hazards such as exposure to exhaust smoke (90.1%) and PM (86.1%) are commonly reported. A significant proportion of MFDRs experience work-related health issues, particularly musculoskeletal disorders (62.1%) and ocular conditions (45.1%). The most frequently reported musculoskeletal complaints include shoulder pain (39.2%) and neck pain (38.1%), while burning eyes (33.3%) was the most common ocular symptom. The findings indicate that prolonged exposure to static postures, repetitive movements, awkward working positions, and whole-body vibration is significantly associated with WRMSDs (*p* < 0.05). Furthermore, psychological hazards demonstrate a significant correlation with symptoms such as headaches, insomnia, and feeling depressed (*p* < 0.05). Physical hazards, including prolonged exposure to sunlight and heat, noise, and whole-body vibrations, are also significantly linked to symptoms such as headaches, flu-like symptoms, and insomnia (*p* < 0.05). Regarding the use of PPE, the most commonly worn items among MFDRs include helmets (99.72%), long-leg pants (99.72%), face masks (99.29%), and thermal jackets (98.17%).

### Biomechanical hazards with work-relatedness musculoskeletal disorders among MFDRs

In this study, WRMSDs are the most common new-onset and/or exacerbated health problems suffered by the respondents, which had a prevalence of two-third of participants. (62.1%) The prevalent was similar and higher than a study conducted in China and Korea which reported the prevalent of MSDs of 55 – 67.9% and 29.3-41.3% respectively [4, 7, 21] However, the prevalence in our research was slightly lower than that in Malaysia which around three-fourth (74.9%) of food delivery riders had suffered from MSDs [22]. The most common affected regions in our study were shoulder (39.2%) and neck pain (38.1%) which were consistent to the findings of Yang’s, Li’s, and Yoo’s [4, 7, 21]. Shoulder pain can cause the highest daily life disturbance and those who suffered were more likely to be absent from work [22]. These health effects could be the results from cumulative traumatic injuries as consequences from the exposure to various physical and biomechanical hazards such as arm-hand vibration, painful positions, repetitive movements [23]. Other factors such as age, gender, riding time and BMI were also related to the disorders [24–26]. Elbow is the region that is least injured by the respondents with only around 5%, however it is significantly associated with ergonomic hazard such as repetitive movements and whole-body vibration. Hand-arm vibration during ride can cause more inclined to grip handles leading to increased static muscle activity in the upper limbs such as arms, elbow, neck, and shoulders [27]. Inappropriate prolonged posture such as excessive elongation of neck could lead to kinematic alterations of body structure such as spine and resulting in more severe MSDs [28, 29].

### Physical hazards and health effects among MFDRs

The prevalence of MFDRs suffered from headache (32.2%) and sleep disturbance (16.9%) in Thailand is quite more common than previous study in Korea (28.5% and 1%) [4]. The possible explanation may be due to longer working hours of Thai MFDRs. Around three-fourths of participants reported experiencing from noise hazards. Traffic noise and motorcycle engine sounds remain inevitable problems leading to problems ranging from annoyance and headaches to sleep disturbances and more severe conditions like ischemic heart disease [30]. Our study supports these findings, showing that noise hazards are associated with headaches and insomnia. The prevalence of ocular symptoms, with no comparable prior study, are more striking than expected. A possible explanation is that these ocular symptoms result from the intense and prolonged use of the eyes, combined with exposure to sunlight and heat, especially during long periods of riding. The results also revealed that only about half of the participants (52.75%) used protective eyewear, which increases their exposure to dust particles, prolonged sunlight, and the risk of dry eyes [31–33].

### Psychological hazards and health effects among MFDRs

In the present study, approximately 30 to 55% of participants reported experiencing work-related stress. This is significantly lower comparing to a study in Korea, with the prevalence (Insufficient rest: 68.3%, Dealing with people: 72.3%, Future uncertainty: 70.8%, Time pressure: 74.3%) and notably lower than the reported rate of 95% from MFDR in China [34]. The differences may be attributed to variations in working habits, norms, and perceptions of riders across these countries.

Work-related stress has a significantly negative impact on both physical and mental well-being of workers [34]. Many platform riders experience significant psychosocial stressors such as dealing with customers, time pressure, income uncertainty, long working hours, unfair customer satisfactions rating, and competitive work environment [4, 35]. Which eventually can lead to increased labor intensity, depression, burnout, and higher turnover rates [36] In this study, we also found that occupational stress is significantly associated with feeling depressed, headaches, and insomnia. Previous research has reported a high prevalence of depressive symptoms and occupational stress among platform workers compared to general population [37]. This is largely due to high job strain and low job security, especially during the COVID-19 outbreak in China, which demonstrated that depression among workers is directly related to work environment stressors [38–40]. Additionally, riders in Thailand tend to work longer hours than their counterparts in other countries, with an average of 55.1 h per week compared to around 42 h for Korean riders [4]. Longer working hours have been significantly associated with occupational stress, depression, fatigue, and poor well-being among employees [38, 41].

### PPE used among MFDRs

The use of PPE during work is crucial for the safety and health well-being MFDRs [17]. In our study, helmets were the most used PPE among participants (99.7%). This is a notable finding, as previous research in Thailand indicated helmet-wearing rates among motorcyclists were only about 30.0–55.0% [42–44]. Facial masks were also another frequent used PPE employed by the MFDRs (99.29%). As mentioned before, facial masks were utilized as PPE, serving to safeguard against not only pollution but also the biological aerosols such as COVID-19 [45]. Therefore, nearly all participants in our study (99.2%) reported wearing facial masks, consistent with findings from studies involving food delivery riders in the Philippines [46] and Vietnam [5], where almost all riders wore masks. Interestingly, apart from face masks, the use of other PPE among participants in our study was higher than reported in previous studies focusing on motorcyclists [42]. One possible explanation is that five out of six commonly used PPE items (long leg pants, helmets, face masks, boots, and thermal jackets), worn by more than 90.0% of the participants, were required by the delivery company’s rules and regulations. The company enforces penalties, including permanent suspension, for riders who fail to comply with these PPE requirements. This highlights the importance of company policies and regulations in promoting rider health, which could be further supported through collaboration with health sectors. However, after the COVID-19 pandemic, the prevalence of some PPE such as facial mask used might be decreased since the protection measures in all sectors (customers, restaurants, and riders) have significantly decrease.

Although gloves were commonly worn by participants, they were not specified in the company regulations. The likely reason is that most riders experience heat and sunlight, as evidenced by our study (91.6%). Riders use gloves to prevent skin burns and soft-tissue injuries while gripping motorcycle handles [47]. In contrast, protective eyewear was less commonly used (52.75%). Eye protection is crucial for motorcyclists as it shields the eyes from debris, dust, and particles, enhances visibility in adverse weather conditions, and provides protection from harmful UV rays. Earplugs were not commonly used, even though approximately three-fourths of participants reported exposure to noise hazards. A possible explanation for this is that most participants preferred using earphones to communicate with restaurants and customers. While prior studies have demonstrated that earplugs can be effective in preventing temporary hearing loss among motorcyclists, the long-term efficacy of earplugs remains a subject of debate [6].

To address occupational hazards and consequences health effects are the challenges topic for MFDRs. As part of the hierarchy of controls, the proposed strategies to mitigate the negative impacts of hazards and prevent their occurrence primarily focus on ‘Administrative Controls’ by the platforms and health sectors and ‘PPE’ by the riders. Administrative controls include enhancing worker awareness through education about hazards and their potential consequences, alongside training on proper posture and techniques to mitigate ergonomic risks. Regulatory measures, such as ensuring regular breaks and job rotation, are recommended to limit prolonged exposure by capping maximum daily working hours. Health surveillance is crucial, with routine health check-ups for workers exposed to hazardous conditions, including lung function tests for air pollution, hearing assessments for noise exposure, and screenings for non-communicable diseases like depression and hypertension. Furthermore, providing PPE through companies and health sectors could help reduce the financial burden on riders. In terms of PPE, promoting and supplying effective yet less commonly used equipment is crucial to reducing the incidence of prevalent symptoms, such as providing protective eyewear to prevent ocular issues.

### Limitations

This study has several limitations. First, since the research was using a cross-sectional design, we could only capture the associations between occupational hazards and health effects, but not the causality. Second, recall bias could have affected the results due to the reliance on self-reported questionnaires. Third, riders with more severe health conditions might be missing from the survey since those severe conditions would restrict them from work, and therefore not fulfilled the inclusion requirement of the study. Since the study was conducted during the COVID-19 pandemic, certain aspects need to be considered, particularly the prevalence of biological hazards and the use of facial masks. The prevalence of biological hazards might be higher in the post-COVID-19 period, while the use of facial masks might have decreased, as protective measures in all sectors (customers, restaurants, and riders) have significantly declined after the COVID-19 period. Future research comparing MFDRs with other delivery groups using different modes of transportation should be conducted to enhance understanding and inform evidence-based interventions in this field. Additionally, longitudinal studies examining health effects over time, along with in-depth analyses of critical issues such as physical assaults, should be prioritized. Furthermore, focusing on significant health effects and emerging issues among MFDRs, such as musculoskeletal disorders, is strongly recommended.

## Conclusion

Most of MFDRs are exposed to physical and chemical hazards, particularly heat/sunlight, exhaustion, smoke, and particulate matter. The most prevalent health issues among respondents are musculoskeletal disorders and eye symptoms, which warrant attention. The findings indicate that prolonged exposure to biomechanical hazards is significantly associated with WRMSDs. Furthermore, psychological hazards demonstrate a significant correlation with symptoms such as headaches, insomnia, and feeling depressed. Physical hazards, including prolonged exposure to sunlight and heat, noise, and whole-body vibrations, are also significantly linked to symptoms such as headaches, flu-like symptoms, and insomnia. Regarding the use of PPE, the most commonly worn items among MFDRs include helmets (99.72%), long-leg pants (99.72%), face masks (99.29%), and thermal jackets (98.17%). These results emphasize the importance of collaboration between health sectors and platform companies to promote the health of gig riders by implementing protective measures for workers. For example, establishing health surveillance, and supplying PPE.

## Supplementary Information

Supplementary Material 1.

## Data Availability

The datasets used and/or analyzed during the current study are only available from the corresponding author on reasonable request.

## Abbreviations

aOR: Adjusted odds ratio
CI: Confidence interval
IQR: Interquartile range
MFDRs: Motorcycle food delivery riders
MSDs: Musculoskeletal disorders
PPE: Personal protective equipment
PM: Particulate matter
SD: Standard deviation
STROBE: Strengthening the reporting of observational studies in epidemiology
WRMSDs: Work-relatedness musculoskeletal disorders
