# Supplementary material for: Assessment of occupational hazards, health effects, and personal protective equipment usage among motorcycle food delivery riders in Thailand: a cross-sectional survey
Source: J Occup Med Toxicol. 2025 May 10;20:13. doi: 10.1186/s12995-025-00460-x (PMC12065142; doi:10.1186/s12995-025-00460-x)
Supplement: Supplementary file 1 — Supplementary Material 1. [file 12995_2025_460_MOESM1_ESM.docx]

Supplementary File 1: Questionnaires

**Consent page:** informing the participant that if he or she clicks continue, means that he has agreed to the consent form.

**Part 1: Socio-demographic Information (11 questions)**

1. Biological Sex
   1. Male
   2. Female
2. Age (years)
3. Education level
   1. Primary education or less
   2. Secondary education
   3. Diploma
   4. Bachelors degree or more
4. Weight (kg)
5. Height (m)
6. Marital status
   1. Single
   2. Marriage
   3. Divorced/Widowed
7. Do you smoke?
   1. No, do not smoke
   2. No, quit smoking
   3. Yes, still smoking
8. Do you drink any alcoholic beverage?
   1. No, do not drink
   2. No, quit drinking
   3. Yes, still drinking
9. In the past year, do you routinely have a health check-up?
   1. None
   2. Yes, the company provides it for me.
   3. Yes, I provide it myself
10. Do you have accident insurance?
    1. None
    2. Yes, the company provides it for me.
    3. Yes, I provide it myself
11. In the past year, do you routinely maintain your vehicle?
    1. Yes, regularly
    2. No maintenance in the past year

**Part 2: Job characteristics (5 questions)**

1. In the past 6 months, on average, how long do you work per day? And how many days per week do you work ?
   1. Provide values in hours per day and day per week. (Calculated as hours per week)
2. How long have you been working as motorcycle food delivery rider or motorcycle taxi?
   1. Provide values in years
3. In the past 6 months, in average, how many orders do you receive in an hour?
   1. Provide values in orders per hour
4. What is your current daily income from delivering food? (average)
   1. Providing values in THB
5. In the past 6 months, what time do you mostly work?
   1. Day time (06.00-18.00)
   2. Night time (18.00-6.00)

**Part 3: Occupational Hazards (14 questions)**

- In the last 6 months, have you encountered any of these occupational hazards while delivering food? (Frequency rating: Always, Usually, Sometimes, Seldom, Never)

**Physical hazards**

1. Noise: Disturbing noise from traffic, engines, or other sources.
2. Whole body vibration: Exposure to whole-body vibration caused by vehicles or the environment.
3. Sunlight and heat

**Chemical hazards**

1. Particulate matter: Exposure to particulate matter from air pollution (such as PM2.5 from wildfires)
2. Smoke from the exhaustion: Exposure to smoke particulary from exhaustion, such as exhaust fumes, engine particles, and traffic-related pollutants.

**Biological hazards**

1. Biological aerosol: Exposure to tiny particles or droplets from sneezing or coughing, particularly in customers or restaurant environments with no protection.

**Biomechanical hazards**

1. Inappropriate posture: For example, caused by the delivery bag placed at the back of the motorcycle seat
2. Remain in the same posture for a long time
3. Repeating same movements

**Psychosocial hazards**

1. Stress from commuting/traffic
2. Stress from customer’s behavior: includes low ratings and negative comments from customers, as well as verbal and physical abuse.
3. Stress from long-shift work: Working beyond the duration of a single shift or 12 hours continuously.
4. Stress from income uncertainty
5. Stress from a highly competitive environment

**Part 4: New onset health effects (22 questions)**

- Have you experienced any of these new health effects or any exacerbation of these existing health problems during the past 6 months? (Rating frequency: Yes, No)

**Musculoskeletal disorders (MSDs)**

- 1. Shoulder pain
  2. Neck pain
  3. Lower back pain
  4. Wrist pain
  5. Upper back pain
  6. Hips and thigh pain
  7. Ankle pain
  8. Knee pain
  9. Elbow pain

**Eyes**

- 1. Burning Eyes: sensation of burning, discomfort or irritation in the eyes.
  2. Itching Eyes: sensation in the eyes that makes you feel the urge to rub or scratch.
  3. Unintended tearing

**Respiratory system**

- 1. Allergies: The presence of sneezing, watery runny nose, itchy skin, hives, and itchy/watery eyes after exposure to pollution, smoke, dust mites, dirt, or pollen, particularly after exposure, or mainly in the morning/at night.
  2. Runny nose: The presence of sneezing, watery mucous from the nose.
  3. Difficulty breathing: Shortness of breath or the feeling of not being able to get enough air.
  4. Chronic cough: Cough that persists for 8 weeks or longer, with or without sputum.
  5. Asthma attack

**Skin**

- 1. Skin burn

**Others**

- 1. Headache
  2. Flu-like symptoms: The presence of fever, fatigue, sorethroat, cough, sneezing and watery runny nose
  3. Insomnia: Difficulty initiating sleep or frequently waking up at night
  4. Feeling depressed: Feeling depressed, hopeless, or having little interest or pleasure in doing things.

**Part 5: Personal Protective Equipment (PPE) (9 questions)**

- During your working period, how often have you used these personal protective equipment items? (Rating frequency: Always, Usually, Sometimes, Seldom, Never)

1. Long leg pants
2. Helmet
3. Face Mask
4. Thermal jacket
5. Trainers/Boots
6. Gloves
7. Sunglasses or wind goggles (protective eyewear)
8. Earplugs
9. Knee, arms, or trunk pads
